# Prevalence of uncorrected refractive error in low-resource high schools in the Free State, South Africa

**DOI:** 10.4102/phcfm.v17i1.4967

**Published:** 2025-09-26

**Authors:** Xolani Nyathela, Urvashni Nirghin, Naimah Ebrahim Khan

**Affiliations:** 1 Department of Optometry, Faculty of Health Sciences, University of KwaZulu-Natal, Durban, South Africa

**Keywords:** uncorrected refractive error, myopia, hyperopia, astigmatism, school-going children, quintiles one to three, Free State

## Abstract

**Background:**

The continuous increase in the prevalence of refractive error (RE) globally, with uncorrected refractive error (URE) having been established as the leading cause of visual impairment (VI) in children, is a public health concern. Previous RE studies in South Africa also indicated a growing prevalence, substantiating the burden on schoolchildren.

**Aim:**

To determine the prevalence of URE among high school learners.

**Setting:**

No-fee-paying high schools in the Free State, South Africa.

**Methods:**

A school-based cross-sectional study design was conducted on 13- to 19-year-old learners through a multistage stratified random method. The examination included an unaided logarith of the minimum angle of resolution (LogMAR) visual acuity test, binocular motor alignment tests, ocular health assessment and cycloplegic autorefraction.

**Results:**

A total of 868 learners consented to participate in this study, with a mean age of 16.4 ± 1.7 years. Male participants accounted for 34.5% (*n* = 299), while female participants accounted for 65.6% (*n* = 569), with the majority being Grade 10 learners. The prevalence of URE stood at 27.1% (*n* = 233), with astigmatism being the most prevalent ametropia, accounting for 22.3% (192 cases), followed by myopia at 15.1% (*n* = 130) and hyperopia at 5.4% (*n* = 46).

**Conclusion:**

A high prevalence of URE, especially among older participants, was established, prompting an urgent concerted intervention from all stakeholders to curb the scourge.

**Contribution:**

This study accentuates the visual situation of vulnerable learners, that is, older children from disadvantaged backgrounds in the Free State, which until this point had not been established.

## Introduction

Refractive error (RE) or ametropia is a state wherein the irregularity in the shape and/or size of the eye causes a defect in light rays focusing on the retina, resulting in blurred vision.^1,2,3^ Blurred vision or the mismatch between the eyeball’s axial length and its optical power is often ‘tolerated’ until daily visual tasks are impeded.^4,5^ However, there is almost no threshold to the ‘tolerance’ of blurred vision, as it has become a norm for many children.^5^ Uncorrected refractive error (URE) is considered the leading cause of visual impairment (VI) and the second most common cause of blindness globally.^1,2,4,6,7,8,9,10,11,12^ Uncorrected refractive error is classified as a public health concern affecting about 12 million school-going children who require good vision up to 80% for learning activities.^1,2,4,6,7,9,11,12,13,14,15^ Furthermore, schoolchildren with URE have heightened cognitive and emotional stress when performing during lessons and/or in dealing with complex visual circumstances.^3^

Among eye diseases, URE was considered the greatest burden of disease, outperforming both cataract and glaucoma.^2,6,7^ This burden is associated with URE’s earlier onset, thus affecting individuals for longer periods compared to cataract and glaucoma.^2,6,7^ Moreover, URE results in poor academic and non-academic performance, poor psychosocial well-being, which causes academic frustration leading to dropping out of school.^2,6,15,16^ The self-belief of being stupid, facilitated by the sense of dependence on their peers for both academic and non-academic activities, gives rise to self-doubt and self-isolation.^2,3,16^ This self-isolation impairs their learning (usually conducted in groups) and their mental health.^2,7^ Limited education because of impeded cognitive and psychosocial development, sense of stupidity and low self-worth hinders children from living their full potential, restricts their economic prospects and adversely impacts their quality of life (QoL).^2,4,6,7,8,9,11,12^ All these catastrophic consequences of URE are rife despite RE being the simplest, most cost-effective diagnosis to make and manage.^7^ Developing nations, such as South Africa, bear the greatest burden of disease, with developing countries in Asia heavily burdened by URE.^12,16,17,18,19^ A 2022 South African study noted that the prevalence of URE stood at 36.3%.^2^ Conversely, about 0.2% of African children are affected by URE.^3^ Table 1 demonstrates the upward trajectory of the prevalence of URE, corroborating the ever-increasing global URE prevalence.

**TABLE 1:** Prevalence of refractive error studies in South Africa and other African countries (cycloplegic refraction).

Author	Region	Sample	Age group (years)	RE prevalence (%)	Astigmatism prevalence (%)	Myopia prevalence (%)	Hyperopia prevalence (%)
Magakwe^2^	Limpopo, South Africa	154	14–18	36.3	13.0	21.4	1.9
Tshivhase et al.^31^	Limpopo, South Africa	297	13–14	13.8	7.5	70.0	18.0
Magakwe^1^	Limpopo, South Africa	337	6–18	20.6	7.4	10.4	2.8
Wajuihian and Hansraj^28^	KwaZulu-Natal, South Africa	1583	13–18	15.0	3.0	7.0	5.0
Naidoo et al.^32^	KwaZulu-Natal, South Africa	4890	5–15	61.4	9.0	3.0	2.0
**Other African RE studies**							
Okenwa-Vincent et al.^33^	Kakanya County, Kenya	2821	14–25	8.7	52.0	25.0	23.0
Asare and Morjaria^12^	Bongo District, Ghana	1705	12–15	1.8	0.6	0.8	0.4
Ezegwui et al.^13^	Enugu, Nigeria	1167	5–15	2.1	0.0	95.8	4.2
Ebri et al.^34^	Cross River State, Nigeria	4241	10–18	7.2	4.2	1.72	1.3

Note: Please see the full reference list of the article Nyathela X, Nirghin U, Ebrahim Khan N. Prevalence of uncorrected refractive error in low-resource high schools in the Free State, South Africa. Afr J Prm Health Care Fam Med. 2025;17(1), a4967. https://doi.org/10.4102/phcfm.v17i1.4967, for more information.

RE, refractive error.

The proliferation of digital devices, accompanied by sedentary lifestyles and the inaccessibility of optometry services for children from disadvantaged backgrounds, prompted the undertaking of this study. To the authors’ knowledge, the prevalence of URE in the Free State province is unknown, unlike in other regions of the country (Table 1). Furthermore, this research sought to study one of the most vulnerable cohorts of schoolchildren. Moreover, older school-going children are more prone to URE than their counterparts.^1,20^ To elucidate further, myopia among high schoolchildren (13–15 years) increased to 12.8% from 1.1% among their primary school counterparts (7–9 years).^21^ Another study highlighted myopia among the 14-year-olds to be 6.3%, which increased to 9.6% among the 15-year-olds.^22^

## Research methods and design

A quantitative, school-based cross-sectional study was undertaken in quintiles (Q)1–Q3 schools of the Free State province of South Africa. The demographic dispensation elected into government sought to redress the education inequalities created by the previous regimes.^23^ The quintile system, which divided schools into five quintiles based on the socioeconomic communities they served, was devised to redress these disparities.^23^ Quintiles one to three (Q1–Q3) cater for children from disadvantaged households, thus classified as no-fee schools, with Q1 considered the poorest of schools in South Africa and receiving a substantial government grant.^23^ These schools were selected based on their vulnerability compared to quintiles four to five (Q4–Q5). The selected schools are not only vulnerable in terms of their socioeconomic status, but in terms of social determinants of health (SDH) and URE as well.

The Free State province is one of the nine South African provinces, located in the centre of the country, spanning an area of 129 825 km^2^.^24^ The study was conducted in the Thabo Mofutsanyane, Motheo (the only metropolitan), Xhariep, and Lejweleputswa districts.^25^ The fifth (Fezile Dabi) district was retrospectively excluded because of stringent measures placed by the education authorities.

### Participants and sample size

High school learners in the Free State aged 13–19 years, corresponding to grades 8 to 12, with no corrective lenses, were eligible to participate. Moreover, a grade two to four anterior chamber angle^26^ graded using the Van Herrick technique was necessary for participation. Participants had to have no diseases or medications that were contraindicated for the stimulation of the parasympathetic system. Signed consent and assent forms were also a prerequisite for participation. The formula to calculate a single population proportion was employed to determine the sample size of the learners, as cited in other prevalence studies. It is indicated below:^16,27,28^

$$n=\frac{(Z1-\alpha {)}^{2}\left(P(1-P)\right)}{d^{2}}$$ [Eqn 1]

The formula is composed of *Z*1 – *α* (the 95% confidence interval) of 1.96, *P* (prevalence determined in a previous South African study^1^) of 0.206, and d (the margin of error, the absolute precision) of 0.05. In consultation with a statistician and using the multistage random sampling technique, 1008 participants from 64 Q1–Q3 high schools across the four districts were invited to participate in this study. To ensure equitable representation, a proportional stratified random sampling method was used, with Q1 schools contributing 40%, Q2 schools 28% and Q3 schools 32% of the sample. Using the 2021 provincial Department of Education schools list, schools were arranged in alphabetical order per quintile, then subjected to a systematic random selection after the sampling interval (kth term) calculation.^25,29,30^ Systematic random sampling from class registers was also undertaken to select an average of three participants from each grade to ensure the representation of all grades.^25^ Systematic random sampling is considered an enhanced method compared to simple random sampling and is recommended when dealing with large populations with available lists (as in this study) because of its ease, inexpensiveness and convenience.^30^ A feasibility study was conducted at a conveniently selected secondary school, which subsequently did not form part of the main study. Data were collated by one researcher.

### Clinical assessment

The examination included unaided visual acuity using the Tumbling E Bailey-Lovie logarith of the minimum angle of resolution (LogMAR) chart (recorded in LogMAR notation). Subsequently, the Hirschberg test at 0.4 m and Cover test at both 4 m and 0.4 m for binocular motor fusion assessment were conducted using a penlight touch, a wolf ball, an occluder and a prism bar. The slit lamp was employed to assess the anterior chamber (AC) angle and the health of the anterior segment, while the posterior segment ocular health assessment was performed with a Welch Allyn direct ophthalmoscope.

Two drops of 1% cyclopentolate were administered in each eye. To allow the drops to be fully effective, a waiting period of about 20 min – 25 min was observed. If pupillary reflexes were still observed after this period, a third drop was administered. Cycloplegia was also considered achieved in the absence of pupillary responses.^1,28,32^ Once complete cycloplegia was attained, participants underwent autorefraction examination using a PlenOptika handheld unit (model nr: MRE0010), which was factory calibrated. Numerous studies have employed this unit among school-going children, including preschoolers, thus demonstrating reliable and accurate measurements of RE.^35,36,37,38^ An average measurement of the participant’s refractive status was displayed on the screen for the researcher to note down after 30 s of measurement for each eye.

The URE diagnoses were made as follows: myopia ≤ −0.50 dioptre sphere (DS), hyperopia ≥ +2.00 DS and astigmatism ≤ −0.75 dioptre cylinder (DC) in at least one eye.^1,39^ The diagnoses were further dissected into mild (−0.50 DS to −3.00 DS), moderate (−3.25 DS to −6.00 DS), and high myopia (< −6.00 DS). Furthermore, hyperopia is classified as mild (+2.00 DS to +3.00 DS), moderate (+3.25 DS to +5.75 DS) and high hyperopia (> +6.00).^1,39^ Astigmatism was also categorised as mild (−0.75 DS to −2.00 DC), moderate (−2.00 DS to −4.00 DC) and high (< −4.00 DC). Astigmatism meridian classifications are with-the-rule (WTR), against-the-rule (ATR), and oblique astigmatism (OA).^40^ Moreover, WTR is the axis correcting minus cylinder 180° ± 30°, ATR 90° ± 30°, while OA is divided into two meridian ranges, that is, 31° to 59° and 121° to 149°.^40^ Although an association between RE and academic performance has been established,^41^ the precise clinical threshold at which RE begins to significantly impact learning remains undefined; hence, all levels of VI were considered.

Also, there were cases of compound RE, that is, compound myopic, hyperopic and mixed astigmatism in either eye; antimetropia and anisometropia between the two eyes – all from the raw data as recorded and picked up by the software. All URE cases, as well as ocular pathology cases, were referred to the nearest public health hospital offering eye care services for further treatment. Once the examination was completed, the participants were provided with the results, and the subsequent steps were briefly explained to them in layman’s terms.

### Data management and analysis

Data were recorded on a recording sheet tailor-made for this study. Data were checked for errors and/or inconsistencies before they were entered into the Statistical Package for Social Sciences (SPSS version 27). In consultation with a statistician, data were analysed using descriptive statistics, distribution tables and proportions. The 95% confidence interval (CI) was calculated using binomial distribution for very low prevalence, and *p* < 0.05 was considered statistically significant based on the Pearson Chi-square test. Both the 95% CI and *p*-value were employed to interpret the results. The one-sample Kolmogorov–Smirnov test was used to assess the age distribution of the participants. The distribution of presenting VA categories was tabulated by gender, age, grades, quintiles, as well as districts. All variables, that is, gender, age, race, quintile and grade, were categorical and were summarised using frequency distribution tables, Odds ratios and proportions.

### Ethical considerations

Ethical clearance to conduct this study was obtained from the University of KwaZulu-Natal Biomedical Research and Ethics Committee (No. BREC/00005522/2023).

## Results

A total of 868 high school learners from 51 quintile one to quintile three schools (Q1–Q3) – that is, 21 Q1 schools, 15 Q2 and 15 Q3 schools – took part in this study, translating to a participation rate of 86.31%. An overwhelming majority of learners were black people (97.7%, *n* = 868), followed by 2.2% (*n* = 19) coloured people and 0.1% (*n* = 1) learner of the white learner. Male participants accounted for 34.5% (*n* = 299), while female participants were the majority at 65.6% (*n* = 569). The mean age of the participants was 16.42 years, and the standard deviation (s.d.) was 1.66 years.

As the one-sample Kolmogorov–Smirnov test concurred that age did not follow the normal curve distribution, the median age was 16 years, and the interquartile range was 15–18 years. The majority of participants were from the Thabo Mofutsanyane district (51.3%, *n* = 445) and from Q1 schools (43.1%, *n* = 375). The Xhariep district (10.1%, *n* = 88) had the least number of participants, while the Motheo and Lejweleputswa districts accounted for 15.2% (*n* = 132) and 23.4% (*n* = 203), respectively. Quintile 2 participants comprised 33.3% (*n* = 289), followed by Q3 schools (23.5%, *n* = 204). Most of the participants (20.3%, *n* = 176) were 17 years old, while the 13-year-olds constituted the smallest (3.3%, *n* = 29) age group. The most represented grade was Grade 10, accounting for 26% (*n* = 226), while the least represented grade 12 (9.9%, *n* = 85).

### Visual acuity

The right eye (72.9% *n* = 633, 95% CI: 12.02–12.39) had 0.7% (*n* = 6) fewer cases of unaided visual acuity (VA_sc_) ≤ 0.2 LogMAR than the left eye (73.6%, *n* = 639, 95% CI: 12.07–12.44). However, the difference between the two eyes was not statistically significant (*p* = 0.805), as both right and left eyes recorded an average VA_sc_ of 0.2 LogMAR. Furthermore, the odds ratio of VA_sc_ ranged from 0.517 (sex) to 0.908 (age), and all variables were associated with VA_sc_ ≤ 0.2 LogMAR as they excluded 1. Also, the logistic regression for all the variables was statistically significant (*p* < 0.05). Table 2 presents the results of the right eye.

**TABLE 2a:** The presentation of unaided VA of the right eyes in LogMAR notation.

Demographics	−0.1 to 0.2			0.3 to 0.5			0.6 to 1.0			1.1 to 1.3			FC to NLP			Total		*p* *
	%	*n*	95% CI	%	*n*	95% CI	%	*n*	95% CI	%	*n*	95% CI	%	*n*	95% CI	%	*n*	
**Sex (*N* = 868)**	-	-	69.9–75.8	-	-	12.5–17.2	-	-	9.2–13.4	-	-	4–16	-	-	1–9	-	-	< 0.001
Male	32.1	242	-	25.8	33	-	19.6	19	-	57.1	4	-	33.3	1	-	34.4	299	-
Female	61.8	391	-	74.2	95	-	80.3	78	-	42.9	3	-	66.7	2	-	65.6	569	-
**Age group (years)**	-	-	-	-	-	-	-	-	-	-	-	-	-	-	-	-	-	0.420
13–15	32.1	203	-	25	32	-	17.5	23	-	42.9	3	-	33.3	1	-	30.2	262	-
16–19	67.9	430	-	75	96	-	80.5	74	-	57.1	4	-	66.7	2	-	69.8	606	-
**Grade**	-	-	-	-	-	-	-	-	-	-	-	-	-	-	-	-	-	< 0.001
8	25.3	160	-	13.3	17	-	12.4	12	-	14.3	1	-	33.3	1	-	-	192	-
9	18.6	118	-	24.2	31	-	16.5	16	-	14.3	1	-	-	-	-	-	166	-
10	24.2	153	-	32.0	41	-	30.9	30	-	28.6	2	-	-	-	-	-	226	-
11	23.1	146	-	21.1	27	-	25.8	25	-	-	-	-	66.7	2	-	-	199	-
12	8.8	56	-	9.4	12	-	14.4	14	-	42.9	3	-	-	-	-	-	85	-

**Total**	**72.9**	**633**	**-**	**14.7**	**128**	**-**	**11.2**	**97**	**-**	**0.8**	**7**	**-**	**0.3**	**3**	**-**	**100**	**868**	**-**

VA, visual acuity; LogMAR, logarith of the minimum angle of resolution; NLP, no light perception; FC, finger count; VKC, vernal keratoconjunctivitis; CI, confidence interval.

* , *p*-value: Chi-square test.

**TABLE 2b:** The presentation of unaided VA of the right eyes in LogMAR notation.

Demographic	Logistic regression		
	Odds ratio	95% CI	*p* *
Sex	0.517	0.369–0.726	< 0.001
Age (years)	0.908	0.828–0.994	0.038
Grade	0.843	0.369–0.726	0.005
Quintile	0.736	0.610–0.889	0.001

CI, confidence interval; LogMAR, logarith of the minimum angle of resolution; VA, visual acuity.

* , *p*-value: Chi-square test.

### Uncorrected refractive error

The distribution of URE is tabulated in Table 3. A total of 27.1% (*n* = 233, 95% CI: 24.2–30.1) presented with URE, which was most prevalent among female participants (71.2%, *n* = 166), 16- to 19-year-olds (72.5%, *n* = 169) and Grade 10 participants (33.9%, *n* = 79). No statistical significance (*p* = 0.17) was observed between URE and age, but gender and grade were statistically significant (*p* = 0.045) each.

**TABLE 3a:** The overall distribution of uncorrected refractive error and ametropia types across sex, age, grade, quintile and district.

Demographic	URE				Myopia				Hyperopia				Astigmatism			
	%	*n*	95% CI	*p* *	%	*n*	95% CI	*p* *	%	*n*	95% CI	*p* *	%	*n*	95% CI	*p* *
Prevalence	27.1	233	24.2–30.1	-	15.1	130	12.9–17.6	-	5.4	46	40–70	-	22.3	192	19.6–25.2	-
**Sex**	-	-	-	0.045	-	-	-	0.007	-	-	-	0.396	-	-	-	0.446
Male	28.8	67	-	-	23.8	31	-	-	28.3	13	-	-	31.8	61	-	-
Female	71.2	166	-	-	76.2	99	-	-	71.7	33	-	-	68.2	131	-	-
**Age group (years)**	-	-	-	0.170	-	-	-	0.228	-	-	-	0.837	-	-	-	0.019
13–15	27.5	64	-	-	25.4	33	-	-	37.0	17	-	-	28.6	55	-	-
16–19	72.5	169	-	-	74.6	97	-	-	63.0	29	-	-	71.4	137	-	-
**Grade**	-	-	-	0.009	-	-	-	0.071	-	-	-	0.467	-	-	-	0.400
8	15.5	36	-	-	13.1	17	-	-	15.2	7	-	-	16.1	31	-	-
9	19.7	46	-	-	18.5	24	-	-	23.9	11	-	-	19.8	38	-	-
10	33.9	79	-	-	32.3	42	-	-	30.4	14	-	-	34.9	67	-	-
11	21.9	51	-	-	23.8	31	-	-	26.1	12	-	-	19.8	38	-	-
12	9.0	21	-	-	12.3	16	-	-	4.3	2	-	-	9.4	18	-	-
**Quintile**	-	-	-	0.042	-	-	-	0.257	-	-	-	0.837	-	-	-	0.029
1	42.1	98	-	-	40.8	53	-	-	45.7	21	-	-	42.2	81	-	-
2	28.8	67	-	-	30.0	39	-	-	19.6	9	-	-	27.6	53	-	-
3	29.2	68	-	-	29.2	38	-	-	34.8	16	-	-	30.2	58	-	-

**Total**	**27.1**	**233**	**-**	**-**	**15.1**	**130**	**-**	**-**	**5.4**	**46**	**-**	**-**	**22.3**	**192**	**-**	**-**

URE, uncorrected refractive error; CI, confidence interval.

* , *p*-value: Chi-square test.

**TABLE 3b:** The overall distribution of uncorrected refractive error and ametropia types across sex, age, grade, quintile and district.

Demographic	Logistic regression											
	URE			Myopia			Hyperopia			Astigmatism		
	OR	95% CI	*p* *	OR	95% CI	*p* *	OR	95% CI	*p* *	OR	95% CI	*p* *
Age	1.099	1.003–1.205	0.043	1.132	1.009–1.269	0.035	0.966	0.808–1.155	0.705	1.076	0.976–1.187	0.141
Sex	1.396	1.007–1.937	0.046	1.792	1.165–2.757	0.008	1.329	0.688–2.566	0.397	1.143	0.811–1.610	0.446
Grade	1.085	0.964–1.220	0.175	1.193	1.030–1.381	0.019	1.007	0.799–1.269	0.954	1.056	0.931–1.197	0.396
Quintile	1.152	0.954–1.391	0.141	1.157	0.917–1.461	0.218	1.155	0.798–1.671	0.444	1.163	0.951–1.421	0.141

Note: Other categories: Anisometropia = 7.3% (*n* = 17); Antimetropia = 2.1% (*n* = 5); Amblyopia = 0.4% (*n* = 1).

URE, uncorrected refractive error; OR, odds ratio; CI, confidence interval.

* , *p*-value: Chi-square test.

The logistic regression of URE prevalence increased by a factor of 1.099 (95% CI: 1.003–1.205) as age increased by one unit. Also, URE increased by a factor of 1.396 (95% CI: 1.007–1.937) for female participants compared with male participants. According to the odds ratio, only the age (older) and sex (female) variables were associated with an increase in URE and were statistically significant (*p* < 0.05). This is because the 95% confidence interval (95% CI) for both variables excluded one.

Astigmatism was the most prevalent type of URE, accounting for 22.3% (*n* = 192, 95% CI: 19.6 to 25.2) cases, with female participants being twice as many (2.14 times), albeit not a significant difference (*p* = 0.446). Moreover, astigmatism was most prevalent among the seniors (71.4%, *n* = 137) and Grade 10 (34.9%, *n* = 67) participants. With-The-Rule astigmatism was the most prevalent in both the right (55.5%, *n* = 91) and left eyes (49.4%, *n* = 86), while oblique astigmatism was the least prevalent in the right (15.2%, *n* = 25) and left eyes (20.7%, *n* = 36). In addition, Against-The-Rule accounted for 29.3% (*n* = 48) in the right eye and 29.9% (*n* = 52) in the left eye.

Myopia ranked as the second most prevalent URE at 15.1% (*n* = 130, 95% CI: 12.9–17.6). Male participants with myopia accounted for 23.9% (*n* = 31). Similarly, myopia was most prevalent among the senior participants, 74.6% (*n* = 97). Also, 6 in 10 learners (60.0%, *n* = 78) from the Thabo Mofutsanyane district presented with myopia, compared with 7.7% (*n* = 10) from the Xhariep district. Similar to the URE logistic regression, myopia was associated with an increase in age, grade and the female sex, all of which were statistically significant (*p* < 0.05). Hyperopia was the least prevalent URE (5.4%, *n* = 46) (95% CI: 40–70), with 7 in 10 female participants (71.71%, *n* = 46) being hyperopic. About two-thirds of hyperopia cases (63.0%, *n* = 29) were recorded among the senior learners. Moreover, hyperopia was more prevalent among Grade 9 to Grade 11 participants (80.4%, *n* = 37). According to the odds ratio, hyperopia and astigmatism were not associated with any of the variables, as the 95% CI included one, and the odds ratios were not statistically significant (*p >* 0.05) for all variables. Importantly, URE or any of the types of ametropia were neither associated with nor statistically significant (*p >* 0.05) for the economic classification of the school (Q1–Q3). Table 4 demonstrates the classification of URE.

**TABLE 4:** The gender classification of the study’s uncorrected refractive error per eye.

Demographic	Right eye								Left eye							
	Mild		Moderate		High		Total		Mild		Moderate		High		Total	
	%	*n*	%	*n*	%	*n*	%	*n*	%	*n*	%	*n*	%	*n*	%	*n*
**Myopia**																
Male	21.7	20	28.6	6	38.5	5	24.6	31	22.6	19	24.00	6	36.4	4	24.2	29
Female	78.3	72	71.4	15	61.5	8	75.4	95	77.4	65	76.00	19	63.6	7	75.8	91
Total	73.0	92	16.7	21	10.3	13	126.0	-	70.0	84	20.83	25	9.2	11	120.0	-
**Hyperopia**																
Male	72.7	8	28.6	2	100.0	1	33.3	11	26.1	6	33.30	2	50.0	1	29.1	9
Female	77.3	17	71.4	5	0.0	-	66.7	22	73.9	17	66.70	4	50.0	1	70.9	22
Total	75.8	25	21.2	7	3.0	1	33.0	-	74.2	23	19.40	6	6.4	2	31.0	-
**Astigmatism**																
Male	33.3	41	42.4	14	18.2	2	6.6	57	31.4	44	31.00	9	50.0	3	6.5	56
Female	66.7	82	57.6	19	81.8	9	12.7	110	68.6	96	69.00	20	50.0	3	13.7	119
Total	14.3	123	3.8	33	1.3	11	167.0	-	16.3	140	3.30	29	0.7	6	175.0	-

### Binocular motor fusion

Heterotropia presented in 2.3% (*n* = 19) and 2.4% (*n* = 20) participants at 4 m and 0.4 m, respectively. Esotropia (1.1%, *n* = 11) was the most prevalent misalignment at 4 m followed by exotropia (0.8%, *n* = 7) and hypotropia associated with exotropia (0.4%, *n* = 2). Similarly, heterotropia at 0.4 m was esotropia (1.1%, *n* = 11), exotropia (0.9%, *n* = 8) and hypotropia associated with exotropia (0.4%, *n* = 2). Statistical significance was observed among those diagnosed with URE and had either latent or manifest deviation at 4 m (*p* = 0.037) and 0.4 m (*p* = 0.001). The only latent deviation detected at 4 m was exophoria (0.5%, *n* = 4) and at 0.4 m was exophoria (36.2%, *n* = 315) as well as esophoria (0.3%, *n* = 3).

### Ocular health

Only two male cases of trauma had pupils responsive only to light and accommodation. Also, one female participant presented with a corneal scar, and 1.9% (*n* = 16) and 1.2% (*n* = 10) had varying forms of corneal opacity in the right and left eyes, respectively. Vernal keratoconjunctivitis (VKC) cases stood at 3.4% (*n* = 29). A total of 0.4% (*n* = 3) presented with glaucomatous signs, and 0.16% (*n* = 1) had optic atrophy in the left eye.

## Discussion

The URE prevalence of 27.1% established in this study in the Free State province is greater than the 20.6%^1^ and 15%^28^ previously established in South Africa, the 16.1% established in Nigeria,^34^ and in many other African studies.^12,13,32^ It is argued that this higher prevalence is not distinctive to the Free State province, but global, as the URE prevalence among school-going children is ever-increasing, given that it has already been classified as either a public health problem or concern.^1,28,39^ This trend is highlighted in a 2022 study carried out elsewhere in South Africa, which found a URE prevalence of 36.3%.^2^ Concerningly, the high prevalence in this study was established among the most vulnerable, that is, mostly learners from lower socioeconomic backgrounds (Q1–Q3) and a higher age cohort. Given the aforementioned, it is possible that these URE cases were long-standing, highlighting a need for routine visual screening, and, where necessary, provision of spectacles at either no cost or at reduced cost, especially for children from low-income backgrounds. Such interventions can reduce health disparities in the country, thus providing children with equal opportunities to progress academically.

Female participants presented with significantly more URE prevalence (19.3%) than their male peers (7.8%), that is, 2.5 times as much. It is possible that the greater participation rate of women, nearly twice as much (1.9x) as their male colleagues, was a contributing factor. However, this finding concurred with several studies,^1,5,28,39^ as women bear the greatest burden of URE because of disadvantaged socioeconomic statuses, especially in low- to middle-income countries.^2,42^ Factors attributed to this phenomenon include biological, cultural (patriarchy), financial (dependence on men) and social (stigmas) reasons.^2,42^ Poor vision associated with URE is more prevalent in senior school learners, indicative of their vulnerability and the need for concerted interventions, as highlighted in this study and other studies^1,22^ Astigmatism was the most prevalent type of ametropia, similar to a Ghanaian study.^3^ It is posited that the high prevalence of astigmatism was exacerbated by the presence of VKC (3.4%), a well-known astigmatism complication.^43^ According to the odds ratio, myopia was the only URE type associated with age, grade and female sex, and was statistically significant (*p* < 0.05) (Table 4). Myopia followed at 15.1%, confirming a more than double increase from 7%^28^ and 10.7%^1^ established in previous South African studies. This increment further substantiates the fact that progressive myopia is a global public health concern, worsened by factors such as sedentary lifestyles, among others.^44,45,46^ Unlike other REs, myopia is complex in that environmental factors like reduced outdoor and/or sunlight exposure periods, sedentary lifestyles, prolonged demand of near vision work, parents with post-matric education, and a myopic parent were noted as predisposing factors.^1,3,4,6,7,9,22,33,34^ It is probable that the factors aforementioned also played a catalytic role in the prevalence established. Both the 2019^34^ and 2020 studies’^1^ prevalence of hyperopia was about half of the 5.4% found in this study, indicative of a growing prevalence of hyperopia.

Eye care services, including vision screening, must have been inaccessible, unavailable, unaffordable, and/or unacceptable for a quarter of the participants to have reduced VA_sc_ associated with URE. This is a clear demonstration of how vulnerable schoolchildren, especially in this setting, are. This reiterates the need for the urgent rollout of eye care services to schoolchildren, given the deleterious effects of compromised vision that extend beyond schooling years. Researchers frequently study the prevalence of RE, whereas this study focused on URE, aiming to highlight how vulnerable learners have become and are in urgent need of eye care. This study alerts eye care practitioners to the situation outside their consultation room and to the need to have every school child have their vision screened, given that South Africa is not spared the ferocious implications of URE. This is because learners, especially those from similar backgrounds, do not actively seek eye care. It is likely that these children unknowingly have had these URE challenges for a while without bringing them to the attention of their parents. The effects of URE are disastrous and have far-reaching implications extending to include the QoL.^2,4,6,7,8,9,11,12^

### Limitation

Stringent terms set by the local authority, compounded by the lack of data collectors, resulted in the retrospective exclusion of the Fezile Dabi district. Notwithstanding the aforementioned, this was a comprehensive study that was representative of the population. Various factors, including no parental consent, withdrawal of assent, and absenteeism, influenced the participation rate. Moreover, Grade 12 learners had to be excluded midway through the study because of examinations that had commenced, resulting in a participation rate of 9.86%, which skewed the results. The study did not measure VI associated URE, as well as the effects of URE on classroom performance.

### Recommendations

A similar study among the same cohort as in this study ought to be undertaken in the Fezile Dabi district because it did not feature in this study. Data collection in similar large-scale research would best be conducted by more than one researcher. Furthermore, studies of this nature need to be conducted throughout the country, given the adverse, life-long effects of URE on children, which have deleterious effects on their education. Barriers to the uptake of spectacles in this population ought to be studied in an attempt to comprehend this prevalence. Future research should aim to integrate objective clinical measures of visual function with academic performance indicators to more precisely evaluate the functional impact of UREs on educational outcomes. This would be crucial, as similar studies were mostly assessed among primary school-going children.^41^ Such an approach would facilitate the identification of clinically significant thresholds of RE that impair learning, thereby informing the development of more targeted and effective interventions within school settings.

## Conclusion

The prevalence of URE in the Free State is considerably higher (27.1%) than in previous similar studies elsewhere in the country (Table 1). This is indicative of the need for urgent collaborative interventions from all stakeholders to provide eye care services to school communities to prevent avoidable blindness on a large scale. The study significantly contributes to the URE database of the province and of the country, especially of older school children who are most susceptible to URE, whose career paths and life potential may be adversely affected by URE.
